# Scabies incidence and association with skin and soft tissue infection in Loyalty Islands Province, New Caledonia: A 15-year retrospective observational study using electronic health records

**DOI:** 10.1371/journal.pntd.0010717

**Published:** 2022-09-06

**Authors:** Yves-Marie Ducrot, Enzo Bruno, Jean-Marc Franco, Loïc Raffray, Samuel Beneteau, Antoine Bertolotti

**Affiliations:** 1 Centre Médico-Social de Wé, Direction de l’Action Communautaire et de l’Action Sanitaire, Province des Iles Loyauté, Lifou, Nouvelle-Calédonie; 2 Département de médecine générale, Université de La Réunion, Saint Pierre, La Réunion, France; 3 Département de médecine interne, CHU de la Réunion, Saint Denis, La Réunion, France; 4 Unité Mixte de Recherche Processus Infectieux en Milieu Insulaire Tropical (UMR PIMIT), Université de La Réunion, CNRS 9192, INSERM 1187, IRD 249, Plateforme Technologique CYROI, Sainte-Clotilde, France; 5 Unité de soutien méthodologique, CHU de la Réunion, Saint Denis, France; 6 Service des Maladies Infectieuses–Dermatologie, CHU de La Réunion, Saint Pierre, La Réunion, France; 7 Inserm CIC1410, CHU de La Réunion, Saint Pierre, La Réunion, France; London School of Hygiene and Tropical Medicine, UNITED KINGDOM

## Abstract

**Background:**

Scabies and its complications are a public health problem in the low- and middle-income countries of the Western Pacific region. However, no data are available for the relatively wealthy French territory of New Caledonia. This study aimed to determine the incidence of scabies and its association with skin and soft tissue infection (SSTI) in Loyalty Islands Province (LIP) (20,000 inhabitants), New Caledonia.

**Methodology/Principal findings:**

This retrospective observational study reviewed cases of scabies and SSTI extracted from the electronic health record databases of LIP clinics for the period 2004–2018. Data were validated through double sampling. The overall scabies incidence rate (IR) and scabies IRs by sex and age group were calculated. Scabies seasonality was evaluated. For children <5 years, the presence of SSTI was compared between the 3-month period preceding scabies diagnosis/treatment and the 3-month period preceding the 1-year anniversary of scabies diagnosis/treatment (self-matching).

A total of 16,843 scabies cases were extracted using a detection algorithm with a sensitivity of 96.7% and a specificity of 99.9%. From 2004 to 2018, the average overall scabies IR was 5.9% and the average scabies IR in children <1 year was 18.4%. Almost two-thirds of children aged 14 years had a history of scabies. Females were more affected, especially in the 20–39 age group (sex ratio>2). A strong seasonality was observed, with a 30% increase in winter. In children <5 years, SSTIs were 4.3 times more frequent in the 3 months preceding the scabies diagnosis than in the 3 months preceding the 1-year anniversary of scabies treatment (p<0.001).

**Conclusions:**

Although health care is much better in New Caledonia than in neighboring countries, scabies is highly endemic in LIP. The disease is especially common in children <2 years and is associated with many SSTIs in children <5 years. Mass drug administration should be considered.

## Author summary

Scabies is a known public health problem in the Pacific region. This study aimed to determine the importance of scabies and associated skin infections in the Loyalty Island Province (LIP) of New Caledonia (20,000 inhabitants).

The provincial electronic medical database was searched for all cases of scabies and skin infection that occurred in the LIP population between 2004 and 2018. To evaluate the impact of scabies on skin infections, we measured the decrease in the number of skin infections in children <5 years one year after scabies treatment.

Over the study period, the average number of scabies cases occurring each year was 6 per 100 inhabitants. In children <1 year, this number was 18 per 100 inhabitants. We found that 80% of children aged 15 years had experienced at least one episode of scabies. We also observed a fourfold decrease in the number of skin infections in children <5 years 1 year after treatment. Scabies was more frequent (+30%) in winter.

## Introduction

Scabies is a skin infestation caused by *Sarcoptes scabiei var*. *hominis* that is transmitted through close skin-to-skin contact and is responsible for pruritic skin rashes. Secondary skin and soft tissue infections (SSTIs) associated with this parasitic disease can be severe and are sometimes complicated by acute post-streptococcal glomerulonephritis or acute rheumatic fever (ARF) [1–4]. Scabies is estimated to affect 200 million people worldwide. In 2017, the World Health Organization (WHO) added scabies to the list of neglected tropical diseases [5].

While scabies and its complications are a known public health problem in the low- and middle-income countries of the Western Pacific region [2], no data are currently available for the relatively wealthy French territory of New Caledonia. However, the Melanesian community, which accounts for half of the population of New Caledonia, has generally low standard of living and suffers a heavy burden of health problems, including severe SSTIs and ARF [6]. One can wonder whether scabies plays a role in the occurrence of these diseases in this population.

The aim of this study was to determine the incidence of scabies and its association with SSTI in Loyalty Islands Province (LIP), New Caledonia.

## Methods

### Ethics statement

The study was approved by the New Caledonia Consultative Ethics Committee (# 202106004) and registered with the French Data Protection Authority (Commission Nationale de l’Informatique et des Libertés, #2211634v0). The study was advertised via posters in all local clinics of LIP.

### Setting

New Caledonia is a French overseas territory (280,000 inhabitants) divided into 3 provinces and located in the Western Pacific between Australia and Fiji. The present study was conducted in LIP, a sparsely populated rural province with 20,000 inhabitants living in 83 small tribes on 3 islands (Ouvéa, Lifou, and Maré). The population of LIP has access to a modern and free public primary and secondary health care system. The density of general practitioners (GPs) is 90 per 100,000 inhabitants, with 93% of the population having access to a GP within a 25-minute drive. The climate is subtropical with 2 marked seasons. The winter is dry and cold and occurs from May to September.

In New Caledonia, scabies is treated in accordance with the guidelines of the French National Authority for Health. Patients receive two doses of: (i) oral ivermectin or (ii) topical benzyl benzoate or (iii) topical permethrin. Simultaneous treatment of all close contacts is also recommended [7,8].

### Study design, sampling, and procedures

This retrospective observational study covered the population attending the local clinics of LIP over the period 2004–2018. These clinics offer care to almost 90% of the LIP population and provide systematic prevention consultations to nearly 100% of LIP children <2 years.

Cases of scabies and SSTI were extracted from the electronic health record (EHR) databases of LIP clinics. These open-source relational databases, which are accessible via an EHR platform software, have been in use in LIP for the past 20 years.

Scabies cases were detected using a Structured Query Language (SQL) algorithm (S1 Text). They were defined as: any medical visit (i) coded as “scabies” (B86) according to the 10^th^ International Classification of Diseases (ICD-10) coding system; (ii) AND/OR with a specific anti-scabies drug recorded in the prescription field or in the free text (iii) AND/OR with the keywords “*gale*” (scab in French) or “scab” recorded in the free text. Two successive visits were classified as separate cases if there was an interval of at least 28 days between them. The algorithm excluded negative formulas (e.g., “not scabies”) and false friends (e.g., “éGALEment”). Some spelling mistakes were allowed. A similar detection algorithm was used for cases of SSTI (S2 Text).

To evaluate the specificity and sensitivity of the detection algorithms, a random draw of 2,714 visits was manually proofread by a physician. This validation approach is considered the gold standard in case detection studies.

The overall incidence rate (IR) of scabies and scabies IRs by sex, age group, and tribe were calculated. In order to assess the cumulative incidence of scabies from 0 to 15 years of age, we extracted the records of 4,732 children <15 years who had completed at least 5 preventive medical visits (mother and child visits, vaccination visits, or school visits) before the age of 5 years, including 1 visit before the age of 1 year. The incidence rate was calculated and averaged for each birth cohort per age group. Only the first episode of scabies was counted in cases of relapse. For children <5 years, the presence of SSTI was compared between the 3-month period preceding scabies diagnosis/treatment and the 3-month period preceding the 1-year anniversary of scabies diagnosis/treatment (self-matching). The latter analysis was restricted to children <5 years who had completed at least 2 preventive medical visits. Scabies seasonality was also evaluated.

Demographic data were obtained by averaging data from the 2004, 2009, 2014, and 2019 population censuses. Climate data were acquired from the meteorological department of New Caledonia.

### Statistical analysis

Incidence rates were calculated after age standardization. Confidence intervals were calculated with a confidence interval of 95% (95% CI). A modified McNemar’s test was used for the analysis of the association between scabies and SSTI in children <5 years. Seasonality was assessed using Pearson correlation coefficient. Statistical analyses were performed with R software version R 4.0.4.

## Results

### Case detection

The detection algorithm for scabies had a sensitivity of 96.7% (95% CI: 90.2–100%) and a specificity of 99.9% (95% CI: 99.8–100%) with a positive predictive value (PPV) of 96.7% and a negative predictive value (NPV) of 99.9%. Overall, 42% of scabies cases were extracted by keyword alone, 13% by prescription alone, and less than 1% by ICD-10 coding alone; moreover, 5% of cases were extracted using the 3 extraction methods (S1 Fig). The performance of the detection algorithm for SSTI was lower, with a sensitivity of 74.6% (95% CI: 69.3–79.9%), a specificity of 99.3% (95% CI: 98.9–99.6%), a PPV of 91.9% and a NPV of 97.4%.

### Incidence of scabies

A total of 16,843 cases of scabies were diagnosed in 10,063 individuals over the 2004–2018 period. The recurrence rate was 36.0%. The annual overall average scabies IR was 5.9%. This IR remained relatively stable over the study period (S2 Fig).

**Fig 1:**
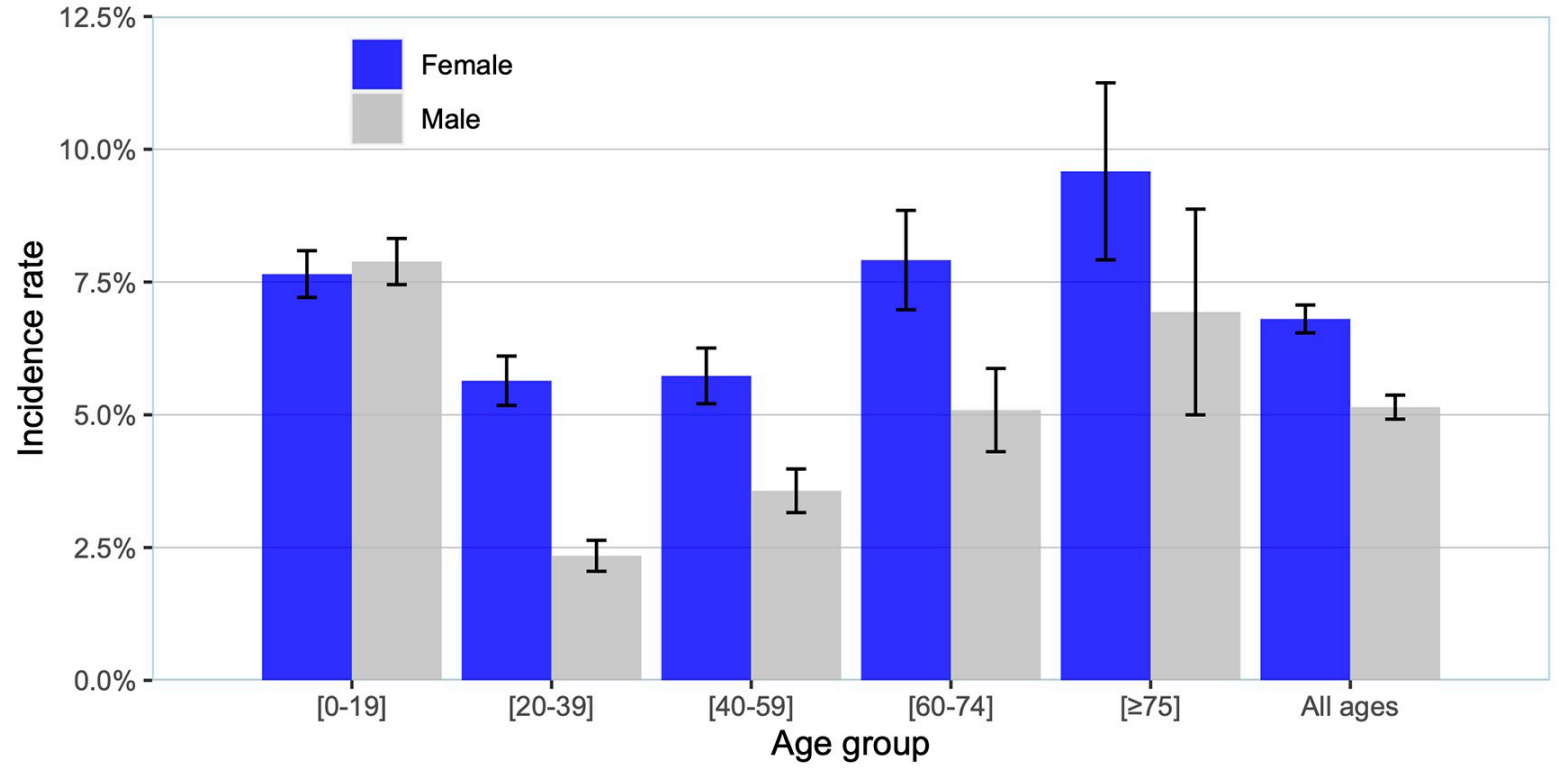
Scabies incidence rate per 1,000 habitants by sex and age group. The histograms represent the average scabies incidence rate in females (blue) and males (grey) according to age group.

The age groups <19 years and >75 years had the highest scabies IRs. The male/female sex ratio (SR) was in favor of females 0.76/1 (95% confidence interval (CI): 0.67, 0.82). In the 20–39 age group, females were twice as affected as males (SR = 0.42/1; 95%CI:0.31,0.53) (Fig 1).

The IR of scabies in children <1 year was 18.4%.

For the entire period of study, no significant differences were observed in the scabies IR between the 3 islands of LIP. However, from 2014 onwards, the scabies IR was higher in Maré than in the other islands (S2 Fig). The scabies IR varied significantly between the different tribes of LIP (S3 Fig).

### Cumulative incidence of scabies

**Fig 2:**
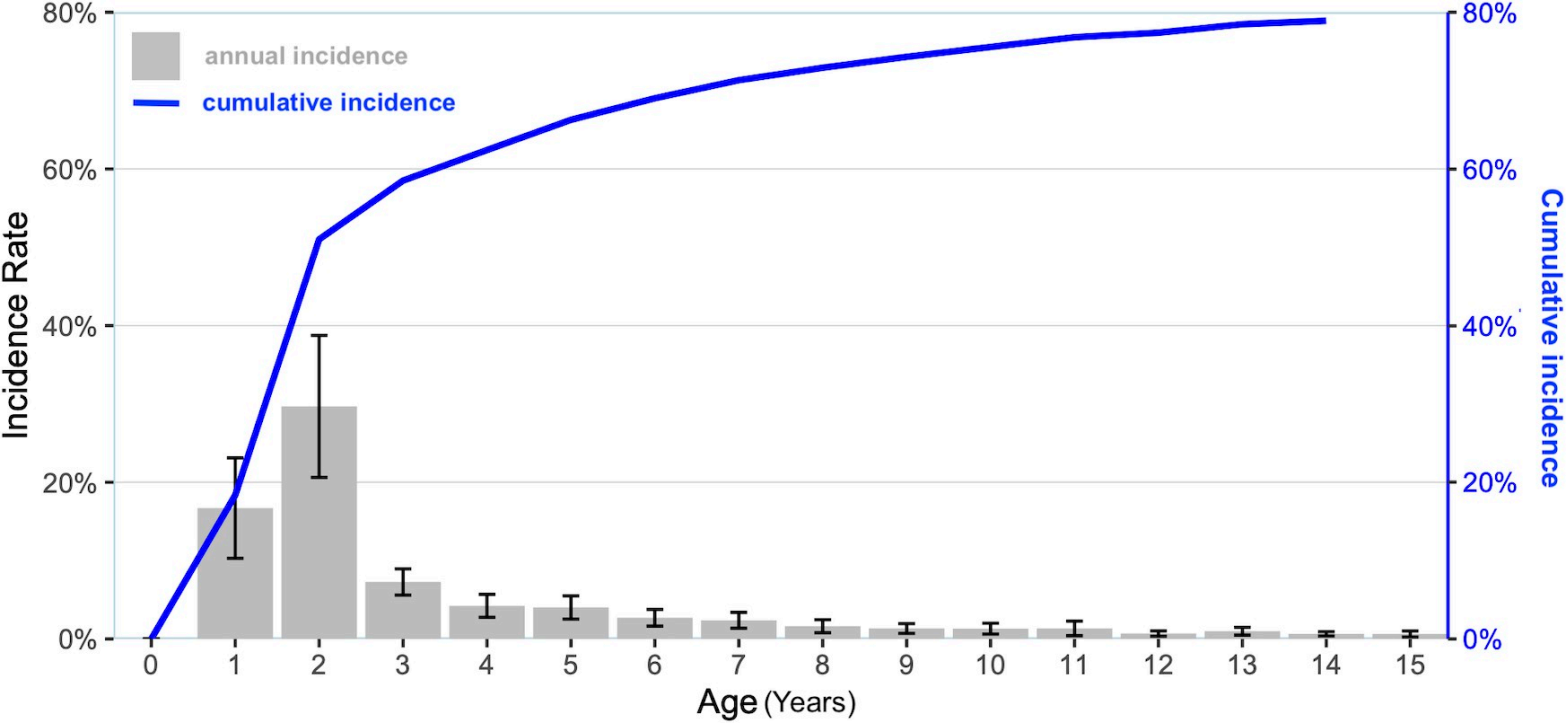
Scabies incidence rate by age group and cumulative incidence from 0 to 15 years. The histogram represents the average scabies incidence rate by age group; the curve represents the cumulative incidence of scabies from 0 to 15 years.

The cumulative incidence of scabies was 51.0% in children aged 2 years, 66.3% in children aged 5 years and 80.2% in children aged 15 years (Fig 2 and S1 Table).

### Seasonality of scabies

**Fig 3:**
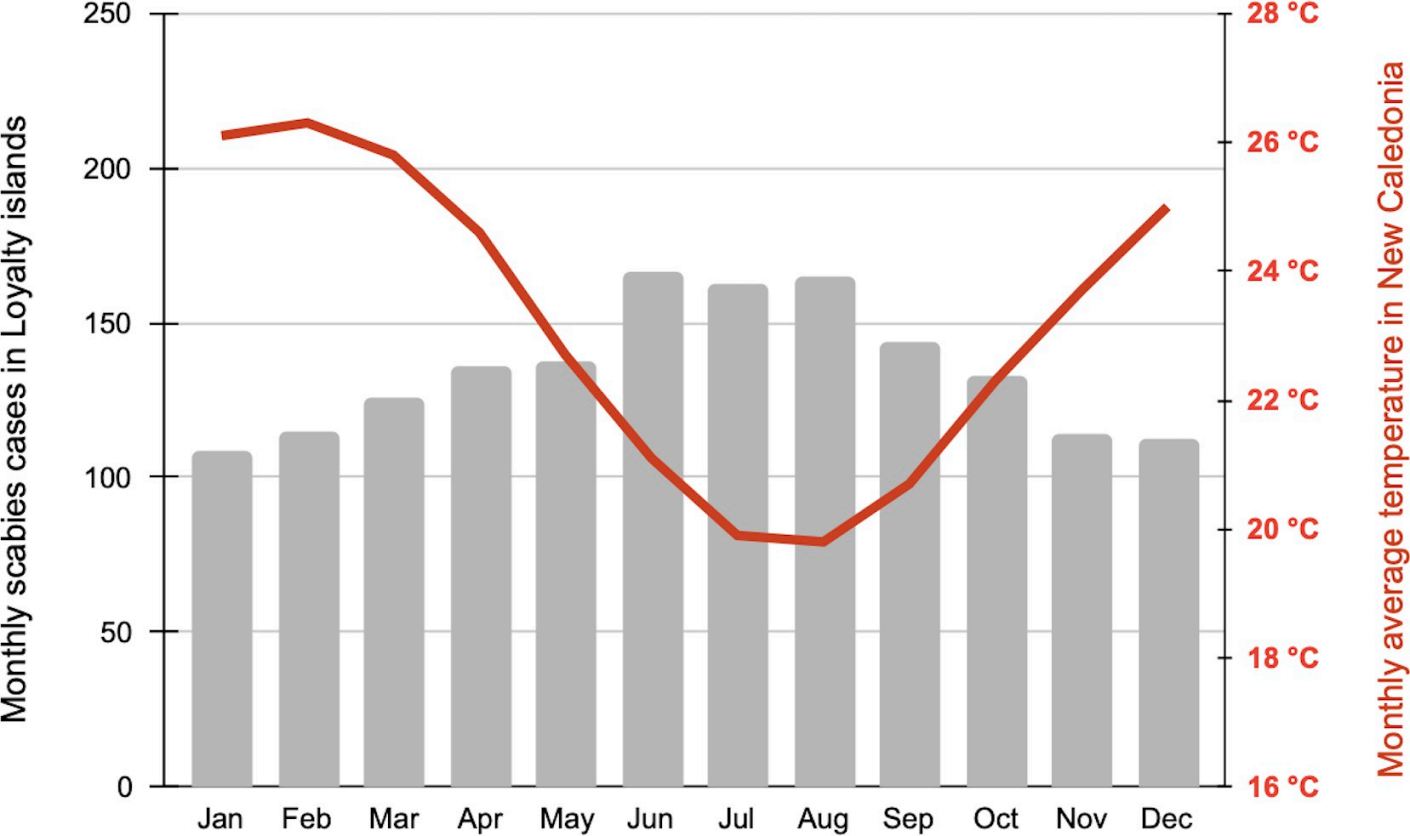
Average monthly number of scabies cases in Loyalty Islands Province from 2004 to 2018 and average monthly temperatures (°C) in New Caledonia from 1981 to 2010. The histogram represents the average monthly number of scabies cases in Loyalty Islands Province (left axis); the curve represents average monthly temperatures (°C) in New Caledonia (right axis).

A statistically significant inverse correlation between monthly scabies cases and monthly temperatures was observed (R = -0.738; p = 0.01), with an increase of nearly 30% between August and January (Fig 3).

### Impact of scabies on skin and soft tissue infections

**Fig 4:**
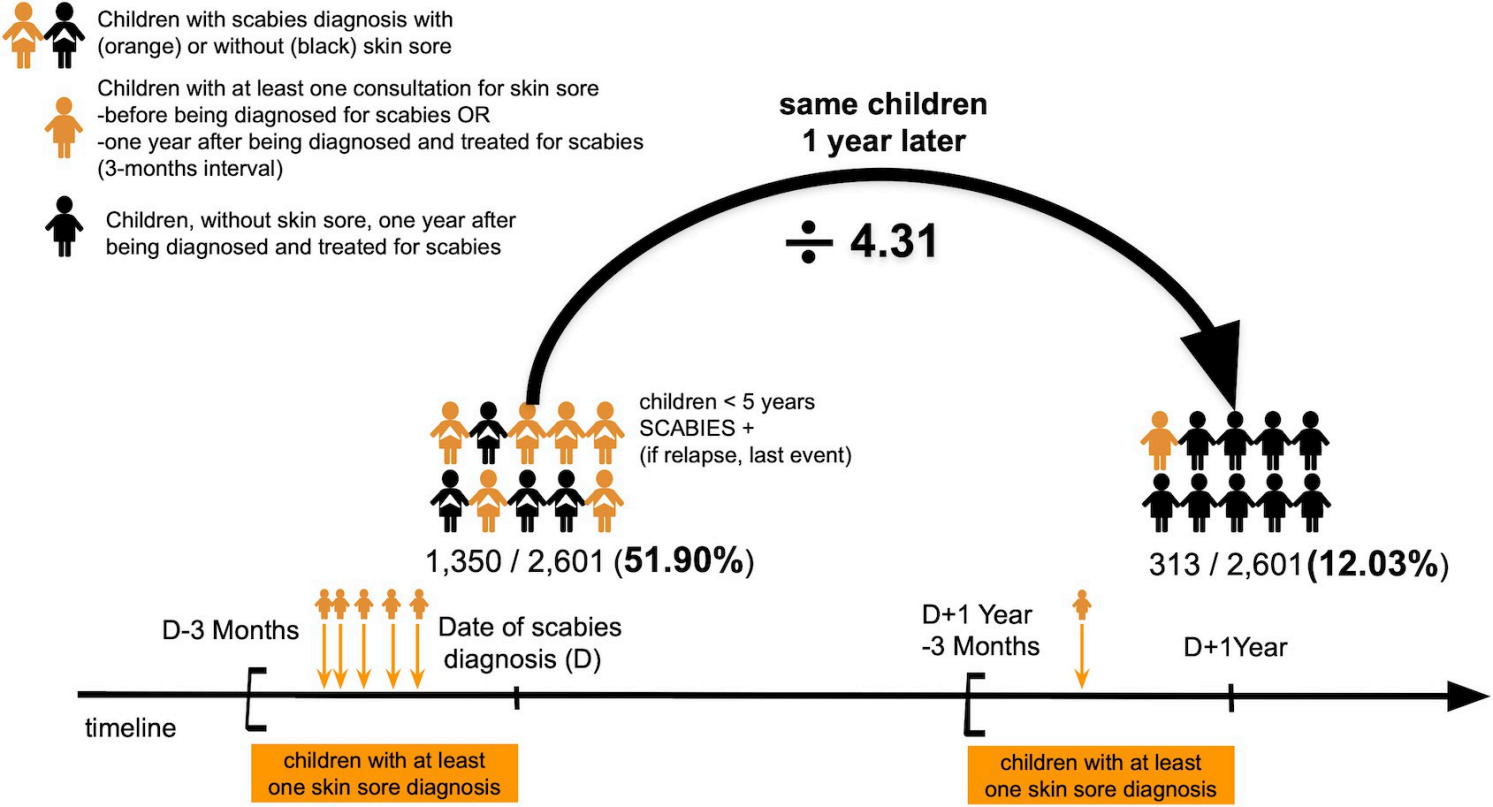
Number of skin and soft tissue infections in children <5 years in the 3 months preceding scabies diagnosis/treatment and in the 3 months preceding the 1-year anniversary of scabies diagnosis/treatment.

In children <5 years, SSTIs were 4.3 times more frequent in the 3 months preceding the scabies diagnosis than in the 3 months preceding the 1-year anniversary of scabies treatment (p<0.001) (Fig 4).

## Discussion

Our study found scabies to be highly endemic in LIP, despite the fact that access to care is much better in New Caledonia than in neighboring countries. Scabies was particularly common in children <2 years and was associated with many SSTIs in children <5 years. It also showed seasonal and geographical variations.

Clinical research based on EHRs is becoming increasingly common in epidemiology [9]. In the absence of systematic IDC coding, cases can be identified by extracting information from the free text of EHRs [10]. While one study in New Zealand did use EHRs from general practices for the detection of SSTI cases [11], to our knowledge our study is the first to use EHRs for the detection of scabies cases.

The overall scabies IR in LIP was 5.9%, a figure 15 to 25 times higher than those reported in European countries [12,13]. Most of the studies conducted in other countries of the Western Pacific have produced data on scabies prevalence, making it difficult to compare the level of scabies endemicity between these and LIP. However, one study conducted in Fiji [14] did report a scabies IR of 2.87% for the 2018–2019 period. While this figure is slightly lower than the IR observed in our study, the level of scabies endemicity is likely to be similar between LIP and Fiji given that these show important differences in care-seeking behavior and access to care.

In our study, the peak of scabies incidence was observed in children aged 2 years. This finding has been reported in one other study [15] that used a longitudinal methodology as we did. Most studies of scabies have found that the disease peaks at the age of 1 year, a phenomenon that is generally explained by a lack of immunity in very young children. The fact that children in New Caledonia are routinely followed up until the age of 2 years could explain the peak of incidence observed at that age in our study.

The SR was strongly in favor of females in our population, especially in the 20–39 age group. Sex imbalance has rarely been reported in the literature on scabies [16]. It could be linked to local cultural practices, as Melanesian mothers generally sleep with their babies. However, it could also reflect a bias due to our use of medical visit data since mothers accompanying children diagnosed with scabies are systematically screened for the disease in New Caledonia.

Our study found a strong seasonality of scabies, with a marked increase in cases between August and January. A similar pattern has been observed in other countries with well-defined seasons [17–19]–one exception being South Korea, where scabies cases have been shown to peak in fall and spring [16]. The recrudescence of scabies in winter can be explained by the increase in promiscuity in cold weather, which in turn favors transmission. Another explanation is that *Sarcoptes* reproduce and survive more easily in fomites with colder temperatures [20].

Despite a very homogeneous lifestyle, significant differences in scabies incidence were found between the tribes of LIP. This phenomenon, which was also observed in the Solomon Islands, has no clear explanation [21] (S3 Fig).

The link between scabies and SSTIs is well known. It is especially well documented for impetigo caused by *Streptococcus pyogenes*, a pathogen that thrives in grooves and scratch lesions as well as in the digestive tract of *Sarcoptes* [22]. To date, however, few studies have quantified the risk of SSTI in patients with scabies [3]. A study using the same methodology as we did, but with a 15-day interval between the occurrence of SSTI and scabies diagnosis/treatment, found an even stronger association [23]. Our study selected a 3-month interval because this corresponds to the peak of natural scabies infestation in humans [24].

In New Caledonia, ARF is especially common in the Melanesian population, with an incidence of 262/100,000 [25,26]. However, unlike the situation in many tropical countries with a high prevalence of ARF, streptococcal angina is rare in New Caledonia [27,28]. The high prevalence of ARF in our population could be explained by the frequent occurrence of impetigo caused by *Streptococcus pyogenes* [29].

Our study has some limitations. The scabies IR could be underestimated as cases were identified based on a presumptive diagnosis that did not follow precise clinical criteria but depended on the physician’s experience. Indeed, GPs working in LIP come mostly from metropolitan France where the scabies IR is low. Given the high GP turnover (the average internship duration is 6 months), it is likely that many cases were missed. Conversely, the scabies IR could be overestimated because asymptomatic contacts are also treated for scabies in New Caledonia. However, the duration and multicentric nature of the study, the large sample size, and the near completeness of data have likely counteracted these biases. We can therefore be fairly confident that the scabies IR in our study is close to the actual IR, particularly among children <2 years. Another limitation is that the 20,000 inhabitants covered in our study represent only 7% of the total population of New Caledonia. Given the variations in socio-cultural and ethnic characteristics across the territory of New Caledonia, our findings cannot be considered representative of other provinces.

Although the population of LIP has access to high-quality healthcare, the scabies IR remained relatively stable in the province over the study period. This finding suggests that more drastic public health measures are needed to reduce the burden of scabies and associated SSTIs in LIP [30–32]. According to a recent WHO framework for scabies control [33,34], ivermectin-based mass drug administration (MDA) should be initiated in areas with a community prevalence **≥**10% or a prevalence in school children ≥ 15% (as measured using a school mapping strategy) and should be avoided in those with a community prevalence <2%. In areas with a community prevalence >2% and <10%, either intensive disease management (IDM) or MDA should be initiated based on an assessment of the local context, as no evidence-based recommendations are currently available to decide on the most appropriate control strategy. Unfortunately, recent data on scabies prevalence in LIP are lacking. Nevertheless, we have seen that the level of scabies endemicity in the province is very close to that in neighboring Fiji [14], where a prevalence of 29% has recently been reported [1]. Accordingly, LIP could meet the WHO threshold for MDA implementation. On the other hand, a 1989 cross-sectional study conducted in a rural area of New Caledonia reported a prevalence of 11% in 269 children <5 years [35]. The relative stability of the scabies IR observed in our study suggests that these prevalence data are still valid today. We can infer from this that either IDM and MDA could be implemented in LIP as per the WHO framework. The first strategy would add little to the management protocol already in place in LIP because it consists mainly in extending treatment to asymptomatic contacts. Mass drug administration, which has shown optimal scabies reduction in small and isolated rural communities with a minimum coverage of 80%, would likely have a greater chance of success as LIP is characterized by insularity, a rural lifestyle, and the presence of small tribes of 50 to 800 inhabitants with a strong political structure. It should be noted, however, that daily aerial exchanges with the large city of Nouméa mitigate this insularity. Moreover, the low vaccination rate against COVID-19 in the province suggests that sufficient MDA coverage would be difficult to achieve. Given these uncertainties, New Caledonia health authorities should consider taking advantage of the ivermectin-based MDA program against filariasis, which is to be launched at the end of 2022 in the smallest island of LIP, to determine the prevalence of scabies and the cost of implementing MDA in all islands of the province.

Despite the fact that access to care is much better in New Caledonia than in neighboring countries, scabies is highly endemic in LIP and is responsible for many SSTIs in children <5 years. Moreover, the scabies IR has remained relatively stable over time, and the recurrence rate is fairly high. Prevalence studies are needed to determine whether more drastic public health measures, including ivermectin-based MDA, must be implemented in LIP to reduce the burden of scabies and associated SSTIs.

## Supporting information

S1 Text Extraction of scabies cases from an open-source SQL relational database management system, using Structured Language Query. (DOCX)

S2 Text Extraction of skin and soft tissue infection cases from an open-source SQL relational database management system, using Structured Language Query. (DOCX)

S1 Fig Venn diagram of scabies case extraction. The blue circle represents the proportion of cases extracted by keyword alone, the green circle the proportion of cases extracted by prescription alone, and the orange circle the proportion of cases extracted by ICD-10 coding alone; the intersection of circles represents the proportion of cases extracted using two or more of these extraction methods.
(TIF)

S2 Fig Evolution of average scabies incidence rates in Loyalty Islands Province and its 3 islands. The histogram represents the average overall incidence rate for the period 2004–2018; the solid curve represents the average incidence rate on the island of Lifou, the large dotted curve represents the average incidence rate on the island of Maré, and the small dotted curve represents the average incidence rate on the island of Ouvéa.
(TIF)

S3 Fig Differences in average incidence rates between the tribes of Loyalty Islands Province. Map created using qGIS, an open source GIS, with two datasets: a) from open source data https://georep-dtsi-sgt.opendata.arcgis.com/datasets/dtsi-sgt::limites-administratives-terrestres-1/explore?layer=1&location=-21.196392%2C165.834400%2C7.88 b) tribes limits was provided by the Information System Department of the Loyalty Island Province.
(TIF)

S1 Data Excel spreadsheet containing, in separate sheets, the underlying numerical data for Figs 1, 2, 3, 4, S1, S2, and S3. (XLSX)

S1 Table Incidence rate for each age group of 14 birth cohort, mean incidence rate and cumulative incidence. (DOCX)
