# Supplementary material for: Scabies incidence and association with skin and soft tissue infection in Loyalty Islands Province, New Caledonia: A 15-year retrospective observational study using electronic health records
Source: PLoS Negl Trop Dis. 2022 Sep 6;16(9):e0010717. doi: 10.1371/journal.pntd.0010717 (PMC9481157; doi:10.1371/journal.pntd.0010717)
Supplement: S1 Text — (DOCX) [file pntd.0010717.s001.docx]

**S1 Text. Extraction of scabies cases from the open-source SQL relational database management system, using Structured Language Query**

| **SELECT**  trim('cim10') as modedd,  evenement.idpatient,  patients.datnaissance,  patients.sexe,  evenement.idevenement ,  evenement.idevenementtype,  evenement.idmedecin,  evenement.datevenement  **FROM** patmaladie  **LEFT JOIN** patients  **ON** patients.idpatient=patmaladie.idpatient  **LEFT JOIN** evenement  **ON** evenement.datevenement=patmaladie.datdeclaration  **AND** evenement.idpatient=patmaladie.idpatient  **LEFT JOIN** ev_consultation  **ON** ev_consultation.idevenement=evenement.idevenement  **WHERE** evenement.datevenement < '2019-01-01'  **AND** evenement.datevenement >= '1999-01-01'  **AND** evenement.idevenementtype  **IN** ('IF0','FI107','IF244','IF245','IF259','IF388','IF49','IF68','IF120','IF268','IF1','IF110','IF243','IF69','IF73','IF74','IF75','IF82')  **AND** patmaladie.idmaladie='B86'  **AND** ev_consultation.idev_consultation <>'NULL'  **UNION ALL**  **SELECT**  trim('libel') as modedd,  evenement.idpatient,  patients.datnaissance,  patients.sexe,  evenement.idevenement ,  evenement.idevenementtype,  evenement.idmedecin,  evenement.datevenement  **FROM** ev_consultation  **LEFT JOIN** evenement  **ON** evenement.idevenement=ev_consultation.idevenement  **LEFT JOIN** patients  **ON** patients.idpatient=evenement.idpatient  **WHERE** evenement.datevenement < '2019-01-01'  **AND** evenement.datevenement >= '1999-01-01'  **AND** evenement.idevenementtype  **IN** ('IF0','FI107','IF244','IF245','IF259','IF388','IF49','IF68','IF120','IF268','IF1','IF110','IF243','IF69','IF73','IF74','IF75','IF82')  **AND (**UPPER(TRIM(evenement.libelle))  **SIMILAR TO** '%GALL?E%'  **AND** UPPER(TRIM(REPLACE(evenement.libelle,'é','e')))  **NOT SIMILAR TO** '%[AUDOE]GALL?E%'  **OR** UPPER(TRIM(evenement.libelle)) **SIMILAR TO** '%SCABI%'  **UNION ALL**  **SELECT**  modedd,  idpatient,  datnaissance,  sexe,  idevenement ,  idevenementtype,  idmedecin,  datevenement  **FROM(SELECT**  trim('ordo') as modedd,  evenement.idpatient,  patients.datnaissance,  patients.sexe,  evenement.idevenement ,  evenement.idevenementtype,  evenement.idmedecin,  evenement.datevenement  **FROM** evenement  **LEFT JOIN** ev_ordonnance  **ON** ev_ordonnance.idevenement=evenement.idevenement  **LEFT JOIN** evenementtype  **ON** evenementtype.idevenementtype=evenement.idevenementtype  **LEFT JOIN** ev_consultation  **ON** ev_consultation.idevenement=evenement.idevenement  **LEFT JOIN** patients  **ON** patients.idpatient=evenement.idpatient  **WHERE** evenement.datevenement < '2019-01-01'  **AND** evenement.datevenement >= '1999-01-01'  **AND** evenement.idevenementtype='IF4'  **AND** UPPER(ev_ordonnance.nomedic )  **SIMILAR TO** '%(SPREGAL\|STROMECTOL\|(A\|TOPI)SCAB)%')  **GROUP BY**  modedd,  idpatient,  datnaissance,  sexe,  idevenement ,  idevenementtype,  idmedecin,  datevenement  **UNION ALL**  **SELECT**  trim('mcttt') as modedd,  evenement.idpatient,  patients.datnaissance,  patients.sexe,  evenement.idevenement ,  evenement.idevenementtype,  evenement.idmedecin,  evenement.datevenement  **FROM** evenement  **LEFT JOIN** evenementtype  **ON** evenementtype.idevenementtype=evenement.idevenementtype  **LEFT JOIN** ev_consultation  **ON** ev_consultation.idevenement=evenement.idevenement  **LEFT JOIN** patients  **ON** patients.idpatient=evenement.idpatient  **WHERE** evenement.datevenement < '2019-01-01'  **AND** evenement.datevenement >= '1999-01-01'  **AND** evenement.idevenementtype  **IN** ('IF0','FI107','IF244','IF245','IF259','IF388','IF49','IF68','IF120','IF268','IF1','IF110','IF243','IF69','IF73','IF74','IF75','IF82')  /*  evenement.idevenementtype='IF0'--cs generaliste  evenement.idevenementtype='FI107'--cs gériatre  evenement.idevenementtype='IF244'--cs urgence  evenement.idevenementtype='IF245'--cs we  evenement.idevenementtype='IF259'--cs scolaire  evenement.idevenementtype='IF388'--cs visite hospit  evenement.idevenementtype='IF49'-- cs pmi  evenement.idevenementtype='IF68'--cs visite dom  */  /*  evenement.idevenementtype='IF120' --cs sf  evenement.idevenementtype='IF268' --cs sf pmi  evenement.idevenementtype='IF1' --ide consult  evenement.idevenementtype='IF110' --ide previsite scolaire  evenement.idevenementtype='IF243' --ide urgence  evenement.idevenementtype='IF69' --ide previsite  evenement.idevenementtype='IF73' --ide pansement simple  evenement.idevenementtype='IF74' --ide pansement lourd  evenement.idevenementtype='IF75' --ide soins à dom  evenement.idevenementtype='IF82' --ide cs pmi  */  **AND**  replace(replace(replace(replace(replace(replace(replace(replace(replace(replace(replace(replace(replace(replace(replace(replace(replace(replace(UPPER((' '\|\|COALESCE(ev_consultation.symptomes,','))\|\|(' '\|\|COALESCE(ev_consultation.diagnostic,','))),'&AGRAVE;','A'),'&ACIRC;','A'),'<BR />','.'),'&NBSP',' '),ASCII_CHAR(13),'.'),ASCII_CHAR(10),'.'),'&EGRAVE;','E'),'&EACUTE;','E'),'&ECIRC;','E'),'&ICIRC;','i'),'&IUML;','i'),'&OCIRC;','O'),'&UGRAVE;','U'),'&UCIRC;','U'),'â','A'),'à','A'),'é','E'),'è','E')  **SIMILAR TO** '%(SPR(E\|A)(I\|Y)GAL\|(IVER\|STRO)MECT\|(A\|TOPI)SCAB(IOL\|))%'  **UNION ALL**  **SELECT**  trim('mcdg+') as modedd,  evenement.idpatient,  patients.datnaissance,  patients.sexe,  evenement.idevenement ,  evenement.idevenementtype,  evenement.idmedecin,  evenement.datevenement  **FROM** evenement  **LEFT JOIN** evenementtype  **ON** evenementtype.idevenementtype=evenement.idevenementtype  **LEFT JOIN** ev_consultation  **ON** ev_consultation.idevenement=evenement.idevenement  **LEFT JOIN** patients  **ON** patients.idpatient=evenement.idpatient  **WHERE** evenement.datevenement < '2019-01-01'  **AND** evenement.datevenement >= '1999-01-01'  **AND** evenement.idevenementtype  **IN(**'IF0','FI107','IF244','IF245','IF259','IF388','IF49','IF68','IF120','IF268','IF1','IF110','IF243','IF69','IF73','IF74','IF75','IF82')  **AND** replace(replace(replace(replace(replace(replace(replace(replace(replace(replace(replace(replace(replace(replace(replace(replace(UPPER((' '\|\|COALESCE(ev_consultation.symptomes,','))\|\|(' '\|\|COALESCE(ev_consultation.diagnostic,','))),'&AGRAVE;','A'),'&ACIRC;','A'),'<BR />','.'),'&NBSP',' '),ASCII_CHAR(13),'.'),ASCII_CHAR(10),'.'),'&EGRAVE;','E'),'&EACUTE;','E'),'&ECIRC;','E'),'&ICIRC;','i'),'&IUML;','i'),'&OCIRC;','O'),'&UGRAVE;','U'),'&UCIRC;','U'),'â','A'),'à','A')SIMILAR TO '%[[:WHITESPACE:].?!:,;=+-/()<>]+(GALL?E\|SCABI(EU\|OS))%'  **UNION ALL**  **SELECT**  trim('mcdg-') as modedd,  evenement.idpatient,  patients.datnaissance,  patients.sexe,  evenement.idevenement ,  evenement.idevenementtype,  evenement.idmedecin,  evenement.datevenement  **FROM** evenement  **LEFT JOIN** evenementtype  **ON** evenementtype.idevenementtype=evenement.idevenementtype  **LEFT JOIN** ev_consultation  **ON** ev_consultation.idevenement=evenement.idevenement  **LEFT JOIN** patients  **ON** patients.idpatient=evenement.idpatient  **WHERE** evenement.datevenement < '2019-01-01'  **AND** evenement.datevenement >= '1999-01-01'  **AND** evenement.idevenementtype  **IN**('IF0','FI107','IF244','IF245','IF259','IF388','IF49','IF68','IF120','IF268','IF1','IF110','IF243','IF69','IF73','IF74','IF75','IF82')  **AND**  replace(replace(replace(replace(replace(replace(replace(replace(replace(replace(replace(replace(replace(replace(replace(replace(UPPER((' '\|\|COALESCE(ev_consultation.symptomes,','))\|\|(' '\|\|COALESCE(ev_consultation.diagnostic,','))),'&AGRAVE;','A'),'&ACIRC;','A'),'<BR />','.'),'&NBSP',' '),ASCII_CHAR(13),'.'),ASCII_CHAR(10),'.'),'&EGRAVE;','E'),'&EACUTE;','E'),'&ECIRC;','E'),'&ICIRC;','i'),'&IUML;','i'),'&OCIRC;','O'),'&UGRAVE;','U'),'&UCIRC;','U'),'â','A'),'à','A')SIMILAR TO '%(PAS \|NI \|SANS )[^.;:,!?+-()=&<>/]{0,40}(GALL?E\|SCABI(EU\|OS))%' |
| --- |
