# Supplementary material for: Scabies incidence and association with skin and soft tissue infection in Loyalty Islands Province, New Caledonia: A 15-year retrospective observational study using electronic health records
Source: PLoS Negl Trop Dis. 2022 Sep 6;16(9):e0010717. doi: 10.1371/journal.pntd.0010717 (PMC9481157; doi:10.1371/journal.pntd.0010717)
Supplement: S2 Text — (DOCX) [file pntd.0010717.s002.docx]

**S2 Text. Extraction of skin and soft tissue infection cases from an open-source SQL relational database management system, using Structured Language Query**

| **--Requetes infections cutanée**  **------------------------------------------**  **---REQUETE PAR CODE CIM10**  **SELECT**  trim('cim10') **as MODEDD**,  trim('XPN'\|evenement.idevenement) **as CMSIDEVNT,**  trim('XPN'\|\|evenement.idpatient) **as CMSIDPAT**,  extract(year from patients.datnaissance)\|\|'-'\|\|substring(100+extract(month from patients.datnaissance) from 2 for 2)\|\|'-'\|\|substring(100+extract(day from patients.datnaissance) from 2 for 2) as DDN,  patients.sexe **as** **SEXE**,  evenement.idevenementtype **as IDEVTYPE,**  evenement.idmedecin **as IDMED,**  extract(year from evenement.datevenement)\|\|'-'\|\|substring(100+extract(month from evnement.datevenement) from 2 for 2)\|\|'-'\|\|substring(100+extract(day from evenement.datevenement) from 2 for 2)\|\|' '\|\|substring(100+extract(hour from evenement.datevenement) from 2 for 2)\|\|':'\|\|substring(100+extract(minute from evenement.datevenement) from 2 for 2)  **as DDE,**  patmaladie.idmaladie as **REM,**  STRLEN**(**ev_consultation.symptomes)+STRLEN(ev_consultation.diagnostic) as **LENCS**  **FROM** patmaladie  **LEFT JOIN** patients  **ON** patients.idpatient=patmaladie.idpatient  **LEFT JOIN** evenement  **ON**  evenement.datevenement=patmaladie.datdeclaration  **AND**  evenement.idpatient=patmaladie.idpatient  **LEFT JOIN** ev_consultation  **ON** ev_consultation.idevenement=evenement.idevenement  **WHERE**  -------------------------------------------------------------------------------------------  --------------------------------condition commune------------------------------  -------------------------------------------------------------------------------------------  --on limite la recherche à l’age au patient né après 1992  **EXTRACT(YEAR FROM patients.datnaissance)>=1992**  --on limite la recherche au patient agé de moins de 26 ans au moment de la cs  --DATEDIFF(year,patients.datnaissance,evenement.datevenement)<26  AND  --on limite la recherche ou pas à une période  evenement.datevenement < '2019-01-01' **AND** evenement.datevenement >= '1999-01-01'  **AND**  --on limite la recherche à certains évenement medicaux et non médicaux  evenement.idevenementtype **IN** ('IF0','FI107','IF244','IF245','IF259','IF388','IF49','IF68','IF120','IF268','IF1','IF110','IF243','IF69','IF73','IF74','IF75','IF82')  -------------------------------------------------------------------------------------------  --------------------------------condition particuliere------------------------------  -------------------------------------------------------------------------------------------  **AND**  --le codeCIM  patmaladie.idmaladie SIMILAR TO **'%(L01\|L02\|L03\|L08\|J34\|A46\|L88\|L73)%'**  --L02 abces cut et furoncle et anthrax  --L88 Ecthyma  --L73 Folliculite bacterienne  --L01 Impetigo  --L01.1 toutes les impétiginisations  --L08 Pyodermite  --L03 Peryonyxis ( panaris)  --A46 Erysipele  --H60.1 Cellulite de l’oreille externe ( annulé car mal utilisé: sutout des otites)  --J34 infection cutanée du nez  **AND**  --pour éliminer les doublons des evenements car un code cim est rattaché à plusieurs evenement  ev_consultation.idev_consultation <>'NULL'  **UNION ALL**  **--RECHERCHE DANS LE LIBELLE**  **SELECT**  trim('libel') **as MODEDD**,  **trim('MAR') as ile,**  **trim('LAR') as cms,**  evenement.idevenement **as IDEVNT,**  evenement.idpatient **as IDPATIENT**,  extract(year from patients.datnaissance)\|\|'-'\|\|substring(100+extract(month from patients.datnaissance) from 2 for 2)\|\|'-'\|\|substring(100+extract(day from patients.datnaissance) from 2 for 2) as DDN,  patients.sexe **as** **SEXE**,  evenement.idevenementtype **as IDEVTYPE,**  evenement.idmedecin **as IDMED,**  extract(year from evenement.datevenement)\|\|'-'\|\|substring(100+extract(month from evenement.datevenement) from 2 for 2)\|\|'-'\|\|substring(100+extract(day from evenement.datevenement) from 2 for 2)\|\|' '\|\|substring(100+extract(hour from evenement.datevenement) from 2 for 2)\|\|':'\|\|substring(100+extract(minute from evenement.datevenement) from 2 for 2)  **as DDE,**  evenement.libelle as **REM,**  STRLEN**(**ev_consultation.symptomes)+STRLEN(ev_consultation.diagnostic) as **LENCS**  **FROM**  ev_consultation  **LEFT JOIN**  evenement  **ON**  evenement.idevenement=ev_consultation.idevenement  **LEFT JOIN** patients  **ON** patients.idpatient=evenement.idpatient  **WHERE**  -------------------------------------------------------------------------------------------  --------------------------------condition commune------------------------------  -------------------------------------------------------------------------------------------  --on limite la recherche à l’age au patient né après 1992  **EXTRACT(YEAR FROM patients.datnaissance)>=1992**  --on limite la recherche au patient agé de moins de 26 ans au moment de la cs  --DATEDIFF(year,patients.datnaissance,evenement.datevenement)<26  AND  --on limite la recherche ou pas à une période  evenement.datevenement < '2019-01-01' **AND** evenement.datevenement >= '1999-01-01'  **AND**  --on limite la recherche à certains évenement medicaux et non médicaux  evenement.idevenementtype IN ('IF0','FI107','IF244','IF245','IF259','IF388','IF49','IF68','IF120','IF268','IF1','IF110','IF243','IF69','IF73','IF74','IF75','IF82')  -------------------------------------------------------------------------------------------  --------------------------------condition particulière------------------------------  -------------------------------------------------------------------------------------------  **AND**  replace(replace(replace(UPPER(TRIM(evenement.libelle)),'é','E'),'è','E'),'î','I') SIMILAR TO '**%(IMPETI\|ABCE\|FURONC\|ANTH?RAX\|ECTH?_MA\|P[YI]ODERM\|FOLL?ICULIT\|PANN?ARI\|PER[IY]ONN?[IY]XIS\|HYPODERMIT\|DHDB\|ER[EIY]S[IY]PEL\|STAPH_LL?OCC?IE)**%'  -- A NOTER QU on pourrait rajouter PHLEGMON ? MAIS DANS TOUS LES CAS IL FAUT ENLEVER DENT/ 17 (N° DES DENTS)/ APICAL/PARO/VEST(IBULAIRE)/GINGIV/PALATIN/GENCIVE/MOLAIRE  **OR**  replace(replace(replace(replace(UPPER(TRIM(evenement.libelle)),'é','E'),'è','E'),'û','U'),'ô','O') **SIMILAR TO '(PLAIE\|LESION\|BOUTON\|PEAU\|PIQURE\|MORSURE\|BLESSURE\|BRULURE\|BOBO\|PIED\|TALON\|MAIN\|DOIGT\|ONGLE\|ORTEIL\|POUCE\|JAMBE\|BRAS\|MB?I(NF)?[ ]?[GD])_+INFECT%'**  **OR**  replace(replace(replace(replace(UPPER(TRIM(evenement.libelle)),'é','E'),'è','E'),'û','U'),'ô','O') **SIMILAR TO 'INFEC_+(PLAIE\|LESION\|BOUTON\|PEAU\|PIQURE\|MORSURE\|BLESSURE\|BRULURE\|BOBO\|PIED\|TALON\|MAIN\|DOIGT\|ONGLE\|ORTEIL\|POUCE\|JAMBE\|BRAS)%'**  **UNION ALL**  **--PAR MEDICAMENT SPECIFIQUE ORAUX DANS L’ORDO INFORMATISE**  **SELECT**  **MODEDD,**  **ile,**  **cms,**  **IDEVNT ,**  **IDPATIENT,**  **DDN,**  **SEXE,**  **IDEVTYPE,**  **IDMED,**  **DDE,**  **REM,**  **LENCS**  **FROM(**  **SELECT**  trim('ordoPO') **as MODEDD**,  **trim('MAR') as ile,**  **trim('LAR') as cms,**  evenement.idevenement **as IDEVNT,**  evenement.idpatient **as IDPATIENT**,  extract(year from patients.datnaissance)\|\|'-'\|\|substring(100+extract(month from patients.datnaissance) from 2 for 2)\|\|'-'\|\|substring(100+extract(day from patients.datnaissance) from 2 for 2) as DDN,  patients.sexe **as** **SEXE**,  evenement.idevenementtype **as IDEVTYPE,**  evenement.idmedecin **as IDMED,**  extract(year from evenement.datevenement)\|\|'-'\|\|substring(100+extract(month from evenement.datevenement) from 2 for 2)\|\|'-'\|\|substring(100+extract(day from evenement.datevenement) from 2 for 2)\|\|' '\|\|substring(100+extract(hour from evenement.datevenement) from 2 for 2)\|\|':'\|\|substring(100+extract(minute from evenement.datevenement) from 2 for 2)  **as DDE,**  ev_ordonnance.wsproductid as **REM,**  STRLEN**(**ev_consultation.symptomes)+STRLEN(ev_consultation.diagnostic) as **LENCS**  **FROM**  evenement  **LEFT JOIN**  --jointure avec la table ordo  ev_ordonnance  **ON**  ev_ordonnance.idevenement=evenement.idevenement  **LEFT JOIN**  --jointure avec la table evenementtype  evenementtype  **ON**  evenementtype.idevenementtype=evenement.idevenementtype  **LEFT JOIN**  --jointure avec la table des consultations  ev_consultation  **ON**  ev_consultation.idevenement=evenement.idevenement  **LEFT JOIN**  --jointure avec la table patients  patients  **ON**  patients.idpatient=evenement.idpatient  **WHERE**  -------------------------------------------------------------------------------------------  --------------------------------condition commune------------------------------  -------------------------------------------------------------------------------------------  --on limite la recherche à l’age au patient né après 1992  **EXTRACT(YEAR FROM patients.datnaissance)>=1992**  --on limite la recherche au patient agé de moins de 26 ans au moment de la cs  --DATEDIFF(year,patients.datnaissance,evenement.datevenement)<26  AND  --on limite la recherche ou pas à une période  evenement.datevenement < '2019-01-01' **AND** evenement.datevenement >= '1999-01-01'  -------------------------------------------------------------------------------------------  --------------------------------condition particuliere------------------------------  -------------------------------------------------------------------------------------------  **AND**  --IF4 etant l’evenement ordonnance  evenement.idevenementtype='IF4'  **AND**  **(--debut de la parenthèse du OU**  --recherche dans les ordonnances les médicaments spécifiques; a noter qu’il n’y a que les nom commercial et donc la DCI peut être utilisé que s’il y a un générique utilisant ce nom.  **UPPER(**ev_ordonnance.forme ) **SIMILAR TO '%(PYOSTACINE\|BRISTOPEN\|CLOXAC\|ORBENINE)%'**  --on utilise la colonne forme qui est plus complete  **OR**  ev_ordonnance.wsproductid IN (**2508,2511,7204,7205,7199,4765,14051,14052,159956**)  **)--fin de la parenthèse du OU**  --2511 bristopen sirop  --2508 birstopen 500 gellule  --7204 fucidine 250 sirop  --7199 fucidine cp 250  --4765 dalacine cp  --14051 pyostacine 250  --14052 pyostacine 500  -- cas particulier de la fucidine ou l’on utilise le code commercial po car on va distinguer les antibo topic des po  **)GROUP BY**  **MODEDD,**  **ile,**  **cms,**  **IDEVNT ,**  **IDPATIENT,**  **DDN,**  **SEXE,**  **IDEVTYPE,**  **IDMED,**  **DDE,**  **REM,**  **LENCS**  **UNION ALL**  **--PAR MEDICAMENT SPECIFIQUE LOCAUX DANS L’ORDO INFORMATISE**  **SELECT**  **MODEDD,**  **ile,**  **cms,**  **IDEVNT ,**  **IDPATIENT,**  **DDN,**  **SEXE,**  **IDEVTYPE,**  **IDMED,**  **DDE,**  **REM,**  **LENCS**  **FROM(**  **SELECT**  trim('ordoLOC') **as MODEDD**,  **trim('MAR') as ile,**  **trim('LAR') as cms,**  evenement.idevenement **as IDEVNT,**  evenement.idpatient **as IDPATIENT**,  extract(year from patients.datnaissance)\|\|'-'\|\|substring(100+extract(month from patients.datnaissance) from 2 for 2)\|\|'-'\|\|substring(100+extract(day from patients.datnaissance) from 2 for 2) as DDN,  patients.sexe **as** **SEXE**,  evenement.idevenementtype **as IDEVTYPE,**  evenement.idmedecin **as IDMED,**  extract(year from evenement.datevenement)\|\|'-'\|\|substring(100+extract(month from evenement.datevenement) from 2 for 2)\|\|'-'\|\|substring(100+extract(day from evenement.datevenement) from 2 for 2)\|\|' '\|\|substring(100+extract(hour from evenement.datevenement) from 2 for 2)\|\|':'\|\|substring(100+extract(minute from evenement.datevenement) from 2 for 2)  **as DDE,**  ev_ordonnance.wsproductid as **REM,**  STRLEN**(**ev_consultation.symptomes)+STRLEN(ev_consultation.diagnostic) as **LENCS**  **FROM**  evenement  **LEFT JOIN**  --jointure avec la table ordo  ev_ordonnance  **ON**  ev_ordonnance.idevenement=evenement.idevenement  **LEFT JOIN**  --jointure avec la table evenementtype  evenementtype  **ON**  evenementtype.idevenementtype=evenement.idevenementtype  **LEFT JOIN**  --jointure avec la table des consultations  ev_consultation  **ON**  ev_consultation.idevenement=evenement.idevenement  **LEFT JOIN**  --jointure avec la table patients  patients  **ON**  patients.idpatient=evenement.idpatient  **WHERE**  -------------------------------------------------------------------------------------------  --------------------------------condition commune------------------------------  -------------------------------------------------------------------------------------------  --on limite la recherche à l’age au patient né après 1992  **EXTRACT(YEAR FROM patients.datnaissance)>=1992**  --on limite la recherche au patient agé de moins de 26 ans au moment de la cs  --DATEDIFF(year,patients.datnaissance,evenement.datevenement)<26  AND  --on limite la recherche ou pas à une période  evenement.datevenement < '2019-01-01' **AND** evenement.datevenement >= '1999-01-01'  -------------------------------------------------------------------------------------------  --------------------------------condition particuliere------------------------------  -------------------------------------------------------------------------------------------  **AND**  --IF4 etant l’evenement ordonnance  evenement.idevenementtype='IF4'  **AND**  **(--debut de la parenthèse du OU**  --recherche dans les ordonnances les médicaments spécifiques; a noter qu’il n’y a que les nom commercial et donc la DCI peut être utilisé que s’il y a un générique utilisant ce nom.  UPPER(ev_ordonnance.forme ) **SIMILAR TO** **'%(MUPI\|BACTRO[BP]AN\|FUCIDINE_+2 [%])%'**  --on utilise la colonne forme qui est plus complete  **OR**  ev_ordonnance.wsproductid IN (7200,7203,8225)  -- cas particulier de la fucidine ou l’on utilise le code commercial po car on va distinguer les antibo topic des po  **)--fin de la parenthèse du OU**  **)GROUP BY**  **MODEDD,**  **ile,**  **cms,**  **IDEVNT ,**  **IDPATIENT,**  **DDN,**  **SEXE,**  **IDEVTYPE,**  **IDMED,**  **DDE,**  **REM,**  **LENCS**  **UNION ALL**  **--RECHERCHE DANS FEUILLE DE PRESCRIPTION ( HOSPIT CMS) DE MED SPE**  **SELECT**  **MODEDD,**  **ile,**  **cms,**  **IDEVNT ,**  **IDPATIENT,**  **DDN,**  **SEXE,**  **IDEVTYPE,**  **IDMED,**  **DDE**  --,certif  -- ce dernier etant un BLOB  --, CAST(SUBSTRING(textecertif FROM (laPoz) FOR 150) AS VARCHAR(300))  -- ce dernier etant l’extrait du BLOB  **FROM**  **(**  **SELECT**  trim('presHOP') **as MODEDD**,  **trim('MAR') as ile,**  **trim('LAR') as cms,**  evenement.idevenement **as IDEVNT,**  evenement.idpatient **as IDPATIENT**,  extract(year from patients.datnaissance)\|\|'-'\|\|substring(100+extract(month from patients.datnaissance) from 2 for 2)\|\|'-'\|\|substring(100+extract(day from patients.datnaissance) from 2 for 2) as DDN,  patients.sexe **as** **SEXE**,  evenement.idevenementtype **as IDEVTYPE,**  evenement.idmedecin **as IDMED,**  extract(year from evenement.datevenement)\|\|'-'\|\|substring(100+extract(month from evenement.datevenement) from 2 for 2)\|\|'-'\|\|substring(100+extract(day from evenement.datevenement) from 2 for 2)\|\|' '\|\|substring(100+extract(hour from evenement.datevenement) from 2 for 2)\|\|':'\|\|substring(100+extract(minute from evenement.datevenement) from 2 for 2)  **as DDE**  ,ev_certificat.certif  --,evenement.libelle  --,position ('PENI' in upper(REPLACE(ev_certificat.certif,'&nbsp;',' '))) as laPoz0  --,maxvalue(position ('PENI' in upper(REPLACE(ev_certificat.certif,'&nbsp;',' ')))-10,1)*1 as laPoz  --,REPLACE(ev_certificat.certif,'&nbsp;',' ') as textecertif  **FROM**  evenement  **LEFT JOIN** patients  **ON** patients.idpatient=evenement.idpatient  **LEFT JOIN** ev_certificat  **ON** evenement.idevenement=ev_certificat.idevenement  **WHERE**  -------------------------------------------------------------------------------------------  --------------------------------condition commune------------------------------  -------------------------------------------------------------------------------------------  --on limite la recherche à l’age au patient né après 1992  **EXTRACT(YEAR FROM patients.datnaissance)>=1992**  --on limite la recherche au patient agé de moins de 26 ans au moment de la cs  --DATEDIFF(year,patients.datnaissance,evenement.datevenement)<26  **AND**  --on limite la recherche ou pas à une période  evenement.datevenement < '2019-01-01' AND evenement.datevenement >= '1999-01-01'  -----------------------------------------------------------------------------------------  --------------------------------condition particulière------------------------------  -------------------------------------------------------------------------------------------  **AND**  evenement.idevenementtype like '%IF371%'  --evenement representant les certificats  **AND**  replace(replace(UPPER(TRIM(evenement.libelle)),'é','E'),'è','E') SIMILAR TO '%PRESC%'  --on limite la recherche au libellé correspondant au prescription hospit  **AND**  **--mots clé contenant des ttt po ou iv spécifique**  UPPER(ev_certificat.certif) SIMILAR TO '**%(P(I\|Y)OSTAC\|PR(I\|Y)ST(I\|Y)NAM\|BRISTOP\|FUCID\|PENI G\|ORBEN\|OXACIL\|VANCO\|DALAC\|CL(I\|Y)NDA)%**'  )  **------------------------------------------------------------------------------------------------------------------------------**  **--------------*******************************************************************************-------------------**  **--------------recherche par mots clés ensuites ( donc requetable par SSMS)---------------------------**  **--------------*******************************************************************************------------------**  **-----------------------------------------------------------------------------------------------------------------------------**  **UNION ALL**  **--RECHERCHE PAR MC : MEDICAMENT SPECIFIQUE ORAUX DANS L OBSERVATION**  **SELECT**  trim('mctttPO') **as MODEDD**,  **trim('MAR') as ile,**  **trim('LAR') as cms,**  evenement.idevenement **as IDEVNT,**  evenement.idpatient **as IDPATIENT**,  extract(year from patients.datnaissance)\|\|'-'\|\|substring(100+extract(month from patients.datnaissance) from 2 for 2)\|\|'-'\|\|substring(100+extract(day from patients.datnaissance) from 2 for 2) as DDN,  patients.sexe **as** **SEXE**,  evenement.idevenementtype **as IDEVTYPE,**  evenement.idmedecin **as IDMED,**  extract(year from evenement.datevenement)\|\|'-'\|\|substring(100+extract(month from evenement.datevenement) from 2 for 2)\|\|'-'\|\|substring(100+extract(day from evenement.datevenement) from 2 for 2)\|\|' '\|\|substring(100+extract(hour from evenement.datevenement) from 2 for 2)\|\|':'\|\|substring(100+extract(minute from evenement.datevenement) from 2 for 2)  **as DDE**  **FROM**  evenement  --jointure avec la table evenementtype  **LEFT JOIN** evenementtype  **ON** evenementtype.idevenementtype=evenement.idevenementtype  --jointure avec la table des consultations  **LEFT JOIN** ev_consultation  **ON** ev_consultation.idevenement=evenement.idevenement  --jointure avec la table patients  **LEFT JOIN** patients  **ON** patients.idpatient=evenement.idpatient  **WHERE**  --------------------------------------------------------------------------------------------  --------------------------------condition commune------------------------------  -------------------------------------------------------------------------------------------  --on limite la recherche à l’age au patient né après 1992  **EXTRACT(YEAR FROM patients.datnaissance)>=1992**  --on limite la recherche au patient agé de moins de 26 ans au moment de la cs  --DATEDIFF(year,patients.datnaissance,evenement.datevenement)<26  AND  --on limite la recherche ou pas à une période  evenement.datevenement < '2019-01-01'  **AND**  evenement.datevenement >= '1999-01-01'  **AND**  --on limite la recherche à certains évenement medicaux et non médicaux  evenement.idevenementtype **IN** ('IF0','FI107','IF244','IF245','IF259','IF388','IF49','IF68','IF120','IF268','IF1','IF110','IF243','IF69','IF73','IF74','IF75','IF82')  /*  evenement.idevenementtype='IF0'--cs generaliste  evenement.idevenementtype='FI107'--cs gériatre  evenement.idevenementtype='IF244'--cs urgence  evenement.idevenementtype='IF245'--cs we  evenement.idevenementtype='IF259'--cs scolaire  evenement.idevenementtype='IF388'--cs visite hospit  evenement.idevenementtype='IF49'-- cs pmi  evenement.idevenementtype='IF68'--cs visite dom  */  /*  evenement.idevenementtype='IF120' --cs sf  evenement.idevenementtype='IF268' --cs sf pmi  evenement.idevenementtype='IF1' --ide consult  evenement.idevenementtype='IF110' --ide previsite scolaire  evenement.idevenementtype='IF243' --ide urgence  evenement.idevenementtype='IF69' --ide previsite  evenement.idevenementtype='IF73' --ide pansement simple  evenement.idevenementtype='IF74' --ide pansement lourd  evenement.idevenementtype='IF75' --ide soins à dom  evenement.idevenementtype='IF82' --ide cs pmi  */  -------------------------------------------------------------------------------------------  --------------------------------condition particuliere------------------------------  -------------------------------------------------------------------------------------------  **AND**  replace(replace(UPPER( ' ' \|\|TRIM(ev_consultation.symptomes)\|\| ' ' \|\|TRIM(ev_consultation.diagnostic)),'&EACUTE;','E'),'&ACIRC;','A')  **SIMILAR TO '%(P(I\|Y)OSTACINE\|PR(I\|Y)ST(I\|Y)NAM(I\|Y)CINE\|BRISTOTEN\|FUCIDINE (PO \|CP\|COMP)\|PENI M\|ORBENINE\|OXACIL)%'**  **UNION ALL**  **--RECHERCHE PAR MC MEDICAMENT SPECIFIQUE LOCAUX DANS L OBSERVATION**  **SELECT**  trim('mctttLOC') **as MODEDD**,  **trim('MAR') as ile,**  **trim('LAR') as cms,**  evenement.idevenement **as IDEVNT,**  evenement.idpatient **as IDPATIENT**,  extract(year from patients.datnaissance)\|\|'-'\|\|substring(100+extract(month from patients.datnaissance) from 2 for 2)\|\|'-'\|\|substring(100+extract(day from patients.datnaissance) from 2 for 2) as DDN,  patients.sexe **as** **SEXE**,  evenement.idevenementtype **as IDEVTYPE,**  evenement.idmedecin **as IDMED,**  extract(year from evenement.datevenement)\|\|'-'\|\|substring(100+extract(month from evenement.datevenement) from 2 for 2)\|\|'-'\|\|substring(100+extract(day from evenement.datevenement) from 2 for 2)\|\|' '\|\|substring(100+extract(hour from evenement.datevenement) from 2 for 2)\|\|':'\|\|substring(100+extract(minute from evenement.datevenement) from 2 for 2)  **as DDE**  **FROM**  evenement  --jointure avec la table evenementtype  **LEFT JOIN** evenementtype  **ON** evenementtype.idevenementtype=evenement.idevenementtype  --jointure avec la table des consultations  **LEFT JOIN** ev_consultation  **ON** ev_consultation.idevenement=evenement.idevenement  --jointure avec la table patients  **LEFT JOIN** patients  **ON** patients.idpatient=evenement.idpatient  **WHERE**  --------------------------------------------------------------------------------------------  --------------------------------condition commune------------------------------  -------------------------------------------------------------------------------------------  --on limite la recherche à l’age au patient né après 1992  **EXTRACT(YEAR FROM patients.datnaissance)>=1992**  --on limite la recherche au patient agé de moins de 26 ans au moment de la cs  --DATEDIFF(year,patients.datnaissance,evenement.datevenement)<26  AND  --on limite la recherche ou pas à une période  evenement.datevenement < '2019-01-01'  **AND**  evenement.datevenement >= '1999-01-01'  **AND**  --on limite la recherche à certains évenement medicaux et non médicaux  evenement.idevenementtype **IN** ('IF0','FI107','IF244','IF245','IF259','IF388','IF49','IF68','IF120','IF268','IF1','IF110','IF243','IF69','IF73','IF74','IF75','IF82')  /*  evenement.idevenementtype='IF0'--cs generaliste  evenement.idevenementtype='FI107'--cs gériatre  evenement.idevenementtype='IF244'--cs urgence  evenement.idevenementtype='IF245'--cs we  evenement.idevenementtype='IF259'--cs scolaire  evenement.idevenementtype='IF388'--cs visite hospit  evenement.idevenementtype='IF49'-- cs pmi  evenement.idevenementtype='IF68'--cs visite dom  */  /*  evenement.idevenementtype='IF120' --cs sf  evenement.idevenementtype='IF268' --cs sf pmi  evenement.idevenementtype='IF1' --ide consult  evenement.idevenementtype='IF110' --ide previsite scolaire  evenement.idevenementtype='IF243' --ide urgence  evenement.idevenementtype='IF69' --ide previsite  evenement.idevenementtype='IF73' --ide pansement simple  evenement.idevenementtype='IF74' --ide pansement lourd  evenement.idevenementtype='IF75' --ide soins à dom  evenement.idevenementtype='IF82' --ide cs pmi  */  -------------------------------------------------------------------------------------------  --------------------------------condition particuliere------------------------------  -------------------------------------------------------------------------------------------  **AND**  replace(replace(UPPER( ' ' \|\|TRIM(ev_consultation.symptomes)\|\| ' ' \|\|TRIM(ev_consultation.diagnostic)),'&EACUTE;','E'),'&ACIRC;','A')  **SIMILAR TO** **'%(MUPI(D\|R)\|FUCIDINE (CREM\|POM)\|BACTROBAN\|(BAIN D_)?HEXOMEDINE( TRANS)?)%'**  **UNION ALL**  **--RECHERCHE PAR MC DIAGNOSTIC EVOQUE DANS L OBSERVATION**  **SELECT**  trim('mcPatho') **as MODEDD**,  **trim('MAR') as ile,**  **trim('LAR') as cms,**  evenement.idevenement **as IDEVNT,**  evenement.idpatient **as IDPATIENT**,  extract(year from patients.datnaissance)\|\|'-'\|\|substring(100+extract(month from patients.datnaissance) from 2 for 2)\|\|'-'\|\|substring(100+extract(day from patients.datnaissance) from 2 for 2) as DDN,  patients.sexe **as** **SEXE**,  evenement.idevenementtype **as IDEVTYPE,**  evenement.idmedecin **as IDMED,**  extract(year from evenement.datevenement)\|\|'-'\|\|substring(100+extract(month from evenement.datevenement) from 2 for 2)\|\|'-'\|\|substring(100+extract(day from evenement.datevenement) from 2 for 2)\|\|' '\|\|substring(100+extract(hour from evenement.datevenement) from 2 for 2)\|\|':'\|\|substring(100+extract(minute from evenement.datevenement) from 2 for 2)  **as DDE**  --replace(replace(replace(replace(replace(replace(replace(replace(replace(replace(replace(replace(replace(replace(UPPER( ' ' \|\|TRIM(ev_consultation.symptomes)\|\| ' ' \|\|TRIM(ev_consultation.diagnostic)),'&AGRAVE;','A'),'&ACIRC;','A'),'&EACUTE;','E'),'&EGRAVE;','E'),'&ECIRC;','E'),'&ICIRC;','i'),'&IUML;','i'),'&OCIRC;','O'),'&UGRAVE;','U'),'&UCIRC;','U'),'<BR />','.'),'&NBSP',' '),ASCII_CHAR(13),'.'),ASCII_CHAR(10),'.')  **--on eleve les retours chariots et nouvelle ligne qu’on transforme en point**  **FROM**  evenement  --jointure avec la table evenementtype  **LEFT JOIN** evenementtype  **ON** evenementtype.idevenementtype=evenement.idevenementtype  --jointure avec la table des consultations  **LEFT JOIN** ev_consultation  **ON** ev_consultation.idevenement=evenement.idevenement  --jointure avec la table patients  **LEFT JOIN** patients  **ON** patients.idpatient=evenement.idpatient  **WHERE**  --------------------------------------------------------------------------------------------  --------------------------------condition commune------------------------------  -------------------------------------------------------------------------------------------  --on limite la recherche à l’age au patient né après 1992  **EXTRACT(YEAR FROM patients.datnaissance)>=1992**  --on limite la recherche au patient agé de moins de 26 ans au moment de la cs  --DATEDIFF(year,patients.datnaissance,evenement.datevenement)<26  AND  **AND**  --on limite la recherche ou pas à une période  evenement.datevenement < '2019-01-01' **AND** evenement.datevenement >= '1999-01-01'  **AND**  --on limite la recherche à certains événement médicaux et non médicaux  evenement.idevenementtype **IN** ('IF0','FI107','IF244','IF245','IF259','IF388','IF49','IF68','IF120','IF268','IF1','IF110','IF243','IF69','IF73','IF74','IF75','IF82')  -------------------------------------------------------------------------------------------  --------------------------------condition particulière------------------------------  -------------------------------------------------------------------------------------------  **AND**  replace(replace(replace(replace(replace(replace(replace(replace(replace(replace(replace(replace(replace(replace(replace(replace(replace(replace(UPPER( ' ' \|\|TRIM(ev_consultation.symptomes)\|\| ' ' \|\|TRIM(ev_consultation.diagnostic)),'&AGRAVE;','A'),'&ACIRC;','A'),'<BR />','.'),'&NBSP',' '),ASCII_CHAR(13),'.'),ASCII_CHAR(10),'.'),'&EGRAVE;','E'),'&EACUTE;','E'),'&ECIRC;','E'),'&ICIRC;','i'),'&IUML;','i'),'&OCIRC;','O'),'&UGRAVE;','U'),'&UCIRC;','U'),'é','E'),'â','A'),'ô','O'),'î','I')SIMILAR TO **'%[[:WHITESPACE:].?!:,;=+-/()<>]+(IMPETIG\|ABCE\|FURONC\|ANTH?RAX\|ECTH?(Y\|I)MA\|P(Y\|I)ODERM\|FOLL?ICULIT\|PANN?ARI\|PER(Y\|I)ONN?(I\|Y)XIS\|DERMO[-]?H(Y\|I)PODERMIT\|ER(E\|I\|Y)S(I\|Y)PEL)%'** |
| --- |
