# Supplementary figures and images for: Scabies incidence and association with skin and soft tissue infection in Loyalty Islands Province, New Caledonia: A 15-year retrospective observational study using electronic health records
Source: PLoS Negl Trop Dis. 2022 Sep 6;16(9):e0010717. doi: 10.1371/journal.pntd.0010717 (PMC9481157; doi:10.1371/journal.pntd.0010717)

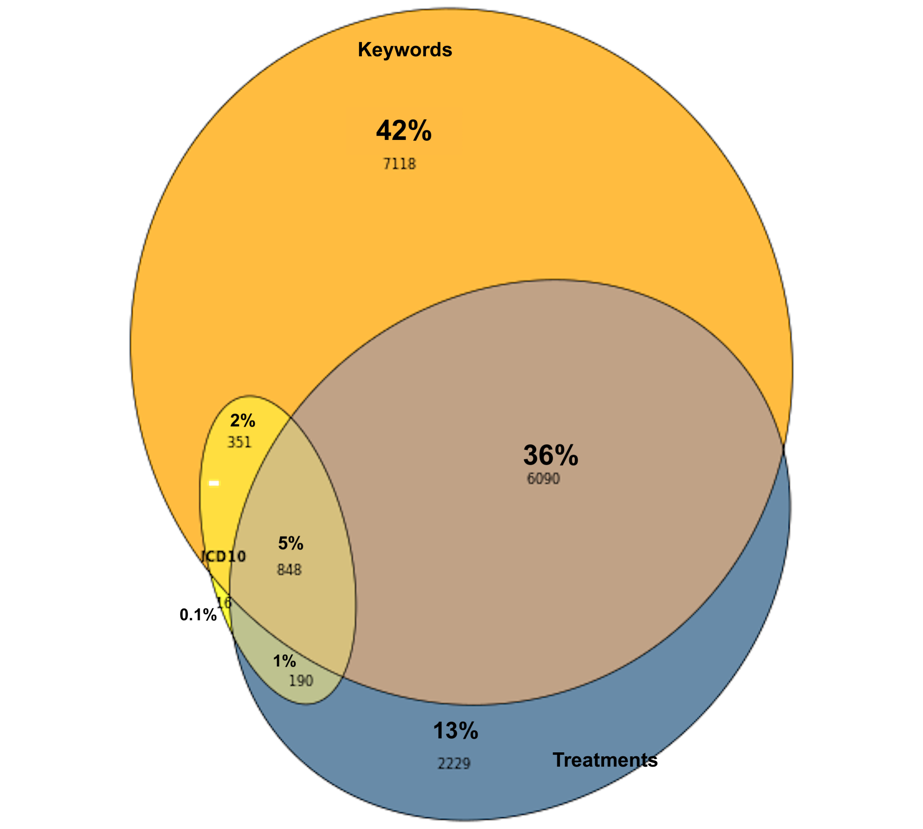

Supplement: S1 Fig — The blue circle represents the proportion of cases extracted by keyword alone, the green circle the proportion of cases extracted by prescription alone, and the orange circle the proportion of cases extracted by ICD-10 coding alone; the intersection of circles represents the proportion of cases extracted using two or more of these extraction methods. (TIF) [file pntd.0010717.s003.tif]

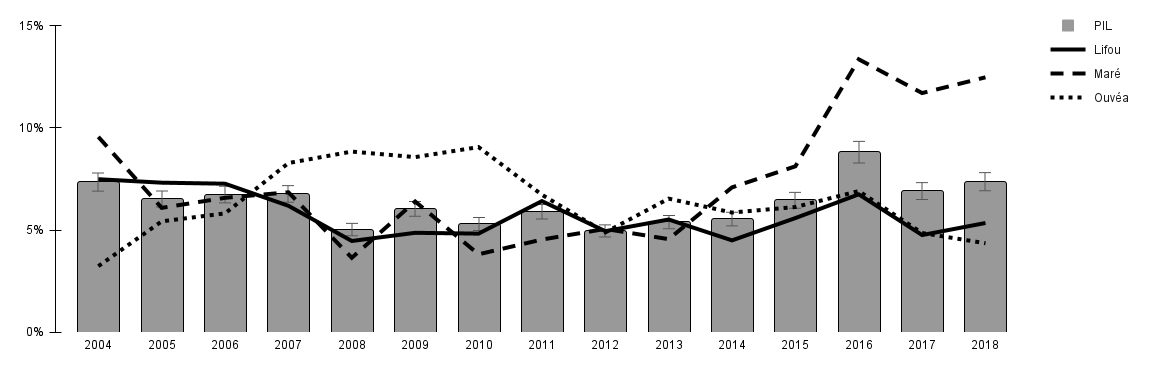

Supplement: S2 Fig — The histogram represents the average overall incidence rate for the period 2004–2018; the solid curve represents the average incidence rate on the island of Lifou, the large dotted curve represents the average incidence rate on the island of Maré, and the small dotted curve represents the average incidence rate on the island of Ouvéa. (TIF) [file pntd.0010717.s004.tif]

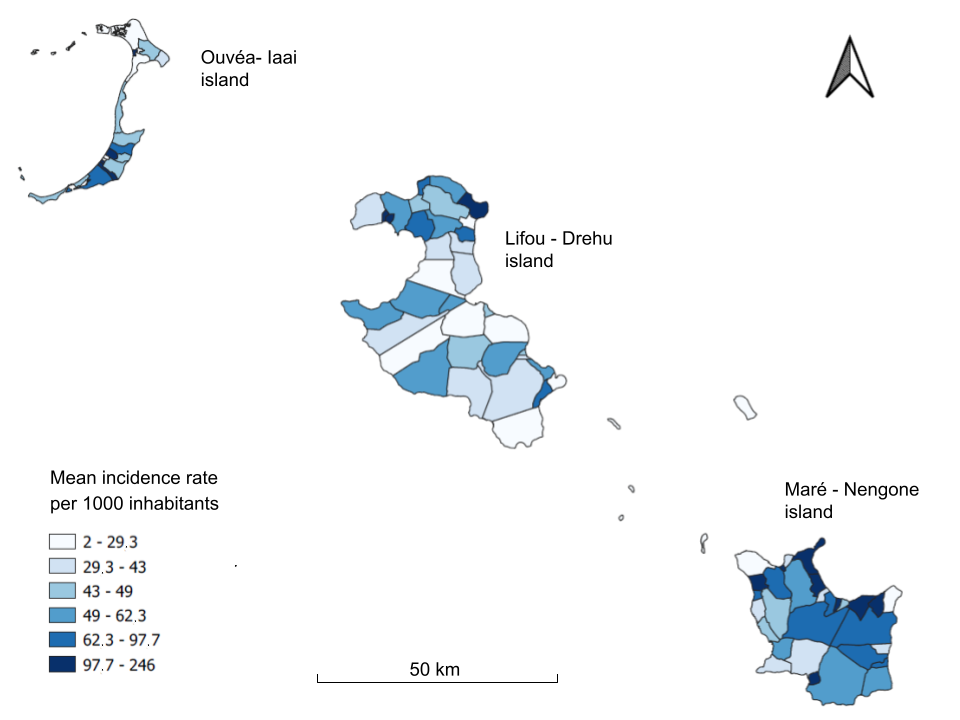

Supplement: S3 Fig — Map created using qGIS, an open source GIS, with two datasets: a) from open source data https://georep-dtsi-sgt.opendata.arcgis.com/datasets/dtsi-sgt::limites-administratives-terrestres-1/explore?layer=1&location=-21.196392%2C165.834400%2C7.88 b) tribes limits was provided by the Information System Department of the Loyalty Island Province. (TIF) [file pntd.0010717.s005.tif]
