# Supplementary material for: Scabies incidence and association with skin and soft tissue infection in Loyalty Islands Province, New Caledonia: A 15-year retrospective observational study using electronic health records
Source: PLoS Negl Trop Dis. 2022 Sep 6;16(9):e0010717. doi: 10.1371/journal.pntd.0010717 (PMC9481157; doi:10.1371/journal.pntd.0010717)
Supplement: S1 Table — (DOCX) [file pntd.0010717.s007.docx]

**S1 Table. Incidence rate for each age group of 14 birth cohort, mean incidence rate and cumulative incidence**
